# The ameliorative effect of ellagic acid on di-(2-ethylhexyl) phthalate-induced testicular structural alterations, oxidative stress, inflammation and sperm damages in adult mice

**DOI:** 10.1186/s12958-021-00830-0

**Published:** 2021-09-18

**Authors:** Azam Hosseinzadeh, Saeed Mehrzadi, Amir Siahpoosh, Zahra Basir, Nosrat Bahrami, Mehdi Goudarzi

**Affiliations:** 1 Razi Drug Research Center, Iran University of Medical Sciences, Tehran, Iran; 2 Medicinal Plant Research Center, Ahvaz Jundishapur University of Medical Sciences, Ahvaz, Iran; 3 Department of Basic Sciences, Faculty of Veterinary Medicine, Shahid Chamran University of Ahvaz, Ahvaz, Iran; 4 Department of Midwifery, Faculty of Nursing and Midwifery, Dezful University of Medical Sciences, Dezful, Iran

**Keywords:** Ellagic acid, Di(2-ethylhexyl) phthalate, Testes, Oxidative stress, Inflammation

## Abstract

**Background:**

Phthalates such as di (2-ethylhexyl) phthalate (DEHP) are well known exogenous substances, disrupting reproductive system function and structure. The current research demonstrated the effect of ellagic acid (EA) on DEHP-induced testicular injury in mice.

**Methods:**

Thirty-five healthy adult male mice were randomly divided to five groups; normal saline receiving group, DEHP (2 g/kg/day, dissolved in corn oil, p.o.) receiving group, DEHP (2 g/kg/day, dissolved in corn oil, p.o.) and EA receiving groups (25, 50 and 100 mg/kg/day, p.o.). Treatment duration of animals was 14 days. Body and testes weights and sperm characteristics and histological changes of testes were evaluated. Serum testosterone, luteinizing hormone (LH) and follicle-stimulating hormone (FSH) levels were analyzed. In the testicular tissue, oxidative/nitrosative stress markers and inflammatory cytokine levels were measured.

**Results:**

Ellagic acid significantly reduced DEHP-induced reduction of body and testes weights. The DEHP-induced reduction of spermatogonia, primary spermatocyte and sertoli cells numbers as well as reduction of sperm vitality and progressive motility were reversed by EA. Furthermore, EA inhibited DEHP-induced alterations in serum hormone levels. These effects were associated with the reduction of DEHP-induced increased level of oxidative stress and inflammatory responses.

**Conclusions:**

Ellagic acid considerably inhibits testicular toxicity of DEHP through reducing oxidative/nitrosative stress and inflammatory responses. Our data suggest that EA may be considered as a promising agent to inhibit male reproductive toxicity induced by endocrine disrupting chemicals such as DEHP.

## Background

Phthalates are well known exogenous substances, which have disrupting effect on reproductive system. These endocrine disrupting chemicals impact male reproductive system through estrogenic and/or anti-androgen properties, which these effects result in the imbalances in hormone homeostasis and subsequent reproductive anomalies [1]. Furthermore, disruption of endocrine system leads to the damage of testicular stem cells compartments resulting in the spermatogenesis disruption, subfertility as well as sperm count reduction [2]. More than 24 types of phthalates exist, which the most important of them include benzyl butyl phthalate (BBP), di-n-butyl phthalate (DnBP), di-iso-nonyl phthalate (DiNP), di-iso-hexyl phthalate (DiHP), diethyl phthalate (DEP) and di(2-ethylhexyl) phthalate (DEHP). Among various types of phthalates, the most male reproductive toxicity is reported for DEHP [3, 4]. This phthalate derivative is frequently used as plasticizer in the manufacture of polyvinyl chloride plastics to render more flexible final products. Unfortunately, DEHP is bound to the matrix of plastic polymer through weak non-covalent links and thus enters into the environment via leaching from plastics [5]. Foods are considered to be the main sources of DEHP in humans [6]. Furthermore, PVC medical devices such as blood storage bags, IV infusion catheters, hemodialysis tubing, syringes and nasogastric tubes insert large amounts of DEHP in the blood and tissues of patients who have had frequent transfusions [7]. The DEHP intake is estimated to be 3–30 μg/kg/day in humans; however, premature neonates in intensive care units receive high exposures to DEHP [1]. Animal studies report that DEHP has hazardous effect on male reproductive system through suppressing testosterone biosynthesis leading to the tubular atrophy and testicular degeneration [1]. DEHP is metabolized to MEHP and 2-ethylhexanol (2-EH) through hydrolyzing enzymes (hydrolysis, unspecific lipases and/or esterases), which are present in various tissues [8]. Furthermore, DEHP has negative impact on pregnancy and increases the risk of developing early embryo loss and congenital malformations [9]. Spermatocyte apoptosis and testicular atrophy induced by MEHP metabolite results from excessive level of oxidative stress; MEHP increases ROS generation and reduces antioxidant levels contributing to the mitochondrial dysfunction and cytochrome c release [10]. Therefore, antioxidant agents seem to be useful to reduce DEHP toxicity in reproductive system [11].

Ellagic acid (EA, C_14_H_6_O_8_) is a naturally-occurring phenolic compound in many natural sources including pomegranates, strawberries, grapes, raspberries and blackberries [12]. Polyphenols are bioactive compounds characterized by one or more hydroxyl groups bonded directly to aromatic phenyl rings. These compounds have various biological properties including antioxidant, anti-inflammatory and anti-carcinogen effects [13]. Similar to other polyphenols, EA exerts antioxidant property through chelating metals, scavenging free radicals and increasing antioxidant enzymes activities; these effects result in the inhibition of reactive oxygen species (ROS)-induced DNA damage and cellular death [14]. Ellagic acid is also effective against inflammation via modulating the expression of inflammatory cytokines such as interleukin (IL)-1β and IL-6. Considering the important etiological role of oxidative stress and inflammation on DEHP-induced male reproductive toxicity and antioxidant and anti-inflammatory properties of EA, it was thought that EA may be a useful agent to reduce DEHP-induced male reproductive toxicity. Current work was therefore carried out to investigate the effects of EA on DEHP-induced testicular injury.

## Materials and methods

### Chemicals

Ellagic acid (EA) and di(2-ethylhexyl) phthalate (DEHP) were purchased from Sigma- Aldrich Chemical Company (St. Louis, MO, U.S.A). All the other reagents were obtained from Merck (Darmstadt, Germany).

### Animals and study design

This study was accepted by Institutional Animal Care and Use Committee of Ahvaz Jundishapur University of Medical Sciences (Ethic code: IR.AJUMS.ABHC.REC.1399.038). Thirty-five healthy adult male NMRI mice (at the age of 4 weeks) were obtained from animal house, Ahvaz Jundishapur University of Medical Sciences, Iran. Animals were kept in groups of three to four/ polycarbonate cages under standard laboratory condition. The temperature of animal room was kept constant at 22 ± 2 °C with 12 h light:12 h dark cycle. Experimental groups included group I receiving normal saline (5 ml/kg/day), group II receiving DEHP (2 g/kg/day, dissolved in corn oil, p.o.), and group III-IV receiving DEHP (2 g/kg/day, dissolved in corn oil, p.o) and EA (25, 50 and 100 mg/kg/day, p.o.). Treatment duration of animals was 14 days.

### Measurement of body and testes weights and Sample collection

In the beginning and at the end of the experiment the body weights of mice were measured. The right and left testes weights were measured at the end of experiment. Blood samples were collected from the heart after anesthetization of animals by injecting ketamine and xylazine (100/10 mg/kg; i.p.) at the end of the treatment. The serum was obtained through centrifuging blood samples (10 min at 1000 x g) and stored at −20 °C for biochemical analysis. Animals were sacrificed by decapitation and testes were removed immediately. Histological examination was done on the left testis and biochemical analysis was done on right testis.

### Serum biochemical parameters

The serum levels of testosterone, LH and FSH were measured using mouse testosterone, LH and FSH ELISA kits (MyBioSource Co, cat num: MBS843463, MBS041300 and MBS2507988, respectively). The values of were expressed as ng/mL for testosterone and mlU/ml for FSH and LH.

### Tissue Biochemical Parameters

The right testis was homogenized (1:10 w/v) in ice-cold Tris-HCl buffer (0.1 M, pH 7.4). After centrifugation (20 min at 4,000 x *g*) at 4 °C, supernatant was collected and stored at −20 °C for biochemical analysis. Bradford method was used to determine total protein concentration of tissue samples.

#### Malondialdehyde (MDA), myeloperoxidase (MPO), protein carbonylation (PC) and nitric oxide (NO·) assay

As an index of lipid peroxidation, the level of MDA was measured in testicular tissue using MDA assay kit (Teb Pazhouhan Razi (TPR), Tehran, IRAN). Further peroxidation in samples was prevented by adding butylated hydroxytoluene (BHT) to tubes containing testicular tissue homogenate. After centrifugation (10 min at 13000 *g*), supernatants of samples were used for analysis. Samples (100 μL) were mixed with detergent (100 μL) and chromogenic solution (thiobarbituric acid, alkali, and acetic acid; 200 μL). Tubes were placed on vigorously boiling water for 1 hour to appear a pink color and then cooled to room temperature. The absorbance of developed pink color was determined at 532 nm using Synergy HT Microplate Reader (BioTek Instruments, Inc, Winooski, VT, USA). The MDA content was determined using MDA standard curve and expressed as nmol/mg protein.

For estimation of inflammatory marker MPO activity in testicular tissue, the tissue homogenates were centrifuged at 4,000 x g for 20 min. The supernatant was discarded and precipitate was re-homogenized in phosphate buffer and 10 mM EDTA. The mixture was centrifuged at 16,000 g at 4 ° C for 10 min. The supernatant was collected and MPO activity was determined by measuring H_2_O_2_-dependent oxidation of o-dianisidine-2HCl. The absorbance was measured at 460 nm for 10 min using a microplate reader (BioTek Instruments, Inc, Winooski, VT, USA). The MPO activity was expressed as unit of MPO/mg protein; 1 unit of MPO was the amount of enzyme caused a change in the absorbance in 1 min at 37 ° C.

The protein oxidation in different groups was determined by measuring the level of protein-bound carbonyl as a biomarker for oxidative stress [15]. To determine the PC level in *testicular tissue*, a mixture of the testis tissue homogenate (0.5 mL) and 0.1% DNPH (w/v in 2 N HCl; 0.5 mL) was prepared and incubated at room temperature for 1 h. For precipitation of total protein, ice-cold 20% TCA (v/v; 1 mL) was added to mixture and centrifuged at 10,000 *g* for 10 min to pellet proteins. The obtained pellet was washed three times *with* ethanol: ethyl acetate (1:1) solution for *removing* the excess unincorporated DNPH. The pellet of samples was dried under a stream of nitrogen and then dissolved in tris buffer solution (1 mL) containing 8.0 M guanidine hydrochloride (pH 7.2). The absorbance was measured using a microplate reader (BioTek Instruments, Inc, Winooski, VT, USA) at 375 nm and expressed as nmol/mg protein.

The level of NO• in testicular tissue was determined using the Griess assay [15]. The supernatant of samples was collected and deproteinized by mixing with zinc sulfate (40 μL, 30% (w/v)). The mixture was centrifuged at 4000 x *g* for 10 min and cadmium granules (2.5- 3 g) were added to mixture. After 2h incubation at room temperature, samples and Griess reagent (5% phosphoric acid containing 0.1% NEDD and 1% sulphanilamide) were mixed in equal volumes. After 10 min incubation at room temperature, the absorbance of samples was measured at 540 nm using Synergy HT Microplate Reader (BioTek Instruments, Inc, Winooski, VT, USA). The NO• level was calculated using sodium nitrite (NaNO2) standard curve and expressed as μmol/mg protein.

#### Antioxidant enzymes activities

The supernatant of samples was used to determine antioxidant enzymes activities. The activity of catalase (CAT) and superoxide dismutase (SOD) was assessed using CAT and SOD assay kits (Teb Pazhouhan Razi (TPR), Tehran, IRAN). To determine CAT activity, the supernatant of samples (20 μl) was mixed with assay buffer (100 μl), methanol (30 μl) and hydrogen peroxide (H_2_O_2_; 20 μl) and incubated in a dark room on a shaker. After 20 min incubation at room temperature, stopper (30 μl) and chromogenic reagents were added to samples and incubated in a dark room on a shaker. After 10 min incubation at room temperature, oxidizer reagent (10 μl) was added to each sample. The plate containing samples was covered and incubated at room temperature for 5 min. The absorbance was measured at 540 nm using a microplate reader (BioTek Instruments, Inc, Winooski, VT, USA). The CAT activity was expressed as units of CAT/mg of protein (U/mg protein).

To determine SOD activity, the supernatants of testicular tissue in different groups were added into each wells of plate. Chromogenic reagent, SOD enzyme solution and SOD assay buffer were then added into wells and the plate was incubated at 37 °C for 20 min. The absorbance of developed yellow color was measured at 450 nm using a microplate reader (BioTek Instruments, Inc, Winooski, VT, USA). The SOD activity was expressed as units of enzyme/mg of protein (U/mg protein).

To determine glutathione peroxidase (GPx) activity, the solution containing NADPH, GR and GSH was added into the wells containing samples of testicular tissues. After 15 min incubation at room temperature, Cumene hydroperoxide solution was added to samples to start the GPx reaction. The absorbance was measured at 340 nm using a microplate reader (BioTek Instruments, Inc, Winooski, VT, USA). The GPx activity was expressed as units of GPx/mg of protein (U/mg protein).

#### The level of glutathione (GSH) and total antioxidant capacity (TAC)

The content of GSH in testicular tissues was determined as previously described [16]. The supernatant of samples was collected and added into the wells containing Ellman's reagent (DTNB; 10 mM in methanol) in Tris–EDTA buffer (pH = 8.6). The mixture was then incubated at room temperature for 20 min to obtain yellow color. The absorbance of obtained color was read at 412 nm using a microplate reader (BioTek Instruments, Inc, Winooski, VT, USA). The GSH standard curve was used to calculate GSH level in each sample, which expressed as nmol/mg protein.

The total antioxidant capacity was determined using assay TAC assay kit (Teb Pazhouhan Razi (TPR), Tehran, IRAN). The supernatant of samples (10 μL) was added to related plate wells. Assay chromogen (150 μL) and assay myoglobin (10 μL) were added to wells containing samples and incubated for 5 min at room temperature. To initiate the reaction, H_2_O_2_ (40 μL) was added to all wells. The plate was covered and incubated on shaker at room temperature for 5 min. Read The absorbance was read at 734 nm using a microplate reader (BioTek Instruments, Inc, Winooski, VT, USA).

#### The level of inflammatory cytokines

The concentrations of TNF-α and IL-1β were measured in the testicular tissue using mouse TNF-α ELISA kit and (Catalog Number: IB49688, IBL company) and mouse IL-1β ELISA kit (Catalog Number: IB99538, IBL company) according to the manufacturer’s protocol and expressed as pg of IL-1β or TNF-α/mg of protein.

### Histological study

The left testis was fixed in Bouin's solution and embedded in paraffin after dehydration.

Sections of tissue were prepared and stained with hematoxylin and eosin (H&E). Twelve cross sections of seminiferous tubules at stages VII-VIII were evaluated in a “blind” fashion under light microscopy (Olympus, Tokyo, Japan) connected to camera (Digital Microscope BMZ-04-DZ) in a magnification 100 X; morphometric parameters including tubular and luminal diameters and epithelial height were assessed using Motic software. The tubules were also assessed for measuring the number of spermatogonia, primary spermatocyte and sertoli cells.

### Sperm analysis

For the evaluation of sperms, the right cauda epididymis was minced and suspended in 1 ml of T6 medium containing 4 mg/ml BSA. After incubation at 37 °C and 5% CO2 for 1 h, 5 μl of the sperm suspension was observed with an optical microscope in a magnification 400 X. Spermatozoon was observed to the evaluation of motility; a total of 100 spermatozoa were analyzed and classified as motile and progressive sperms. For the evaluation of sperm vitality, sperms were stained with eosin B (0.5% in normal saline). In each mouse, a total of 100 spermatozoa were analyzed and classified as motile living sperm, immotile living sperm or dead sperm.

### Statistical analysis

Statistical analysis was performed using Graph Pad Prism version 6 for Windows (Graph Pad Software, USA). Results were presented as mean ± standard deviations (SD). Individual differences between the groups were determined using one-way ANOVA followed by Tukey’s post hoc test and *p* < 0.05 was considered statistically significant.

## Results

### Effects of EA on DEHP-induced alterations in body and testes weights

Exposure of mice with DEHP resulted in the significant reduction of body and testes weights compared to the control group (*p* < 0.05). Administration of EA in 50 and 100 mg/kg could significantly inhibit the reduction of body weight induced by DEHP in mice (*p* < 0.05), but EA in 25 mg/kg dose did not change DEHP-induced reduction of body weight. Treatment with EA did not significantly change testes weights in 25 and 50 mg/kg. Only significant improvement observed in the left testes weight treated with 100 mg/kg EA (*p* < 0.05; Table 1).

**Table 1:** Body and testis weights

	NS	DEHP	DEHP + EA 25	DEHP + EA 50	DEHP + EA 100
Initial body weight(g)	24.2 ± 1.4	23.3 ± 1.2	23.5 ± 1.6	24.1 ± 0.9	22.8 ± 1.5
Final body weight(g)	27.5 ± 1.7	22.8 ± 0.9 ^a^	24.6 ± 1.1 ^a^	26.9 ± 0.8 ^b, c^	26.7 ± 1.2 ^b, c^
Right testis weight(mg)	78.2 ± 6.2	62.3 ± 4.3 ^a^	66.1 ± 5.4 ^a^	67.3 ± 4.8 ^a^	70.4 ± 5.5
Left testis weight(mg)	76.5 ± 6.4	63.7 ± 4.6 ^a^	65.7 ± 6.1 ^a^	66.8 ± 4.6 ^a^	72.3 ± 5.1 ^b^

Values are means ± S.D. (*n* = 7)

^a^*p*<0.05 as compared to control group

^b^*p*<0.05 as compared to DEHP group

^c^*p*<0.05 as compared to DEHP + EA 25 group

### Effects of EA on DEHP-induced alterations in serum testosterone, LH and FSH levels

**Fig. 1:**
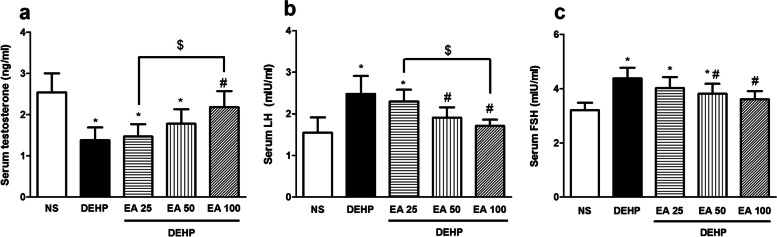
The effect of EA on serum testosterone, LH and FSH levels in mice exposed to DEHP. Values are means ± S.D. (*n* = 7). *p < 0.05 as compared to control group. ^#^*p* < 0.05 as compared to DEHP group

In mice exposed to DEHP, the serum testosterone level significantly decreased (*p* < 0.05) and serum LH and FSH level significantly increased (*p* < 0.05) compared to the control group. Administration of EA in the dose of 100 mg/kg significantly inhibited DEHP-induced alterations in testosterone level. Furthermore, the doses of 50 and 100 mg/kg of EA inhibited DEHP-induced elevation of LH and FSH levels (*p* < 0.05; Fig. 1).

### Effects of EA on DEHP-induced oxidative stress

**Fig. 2:**
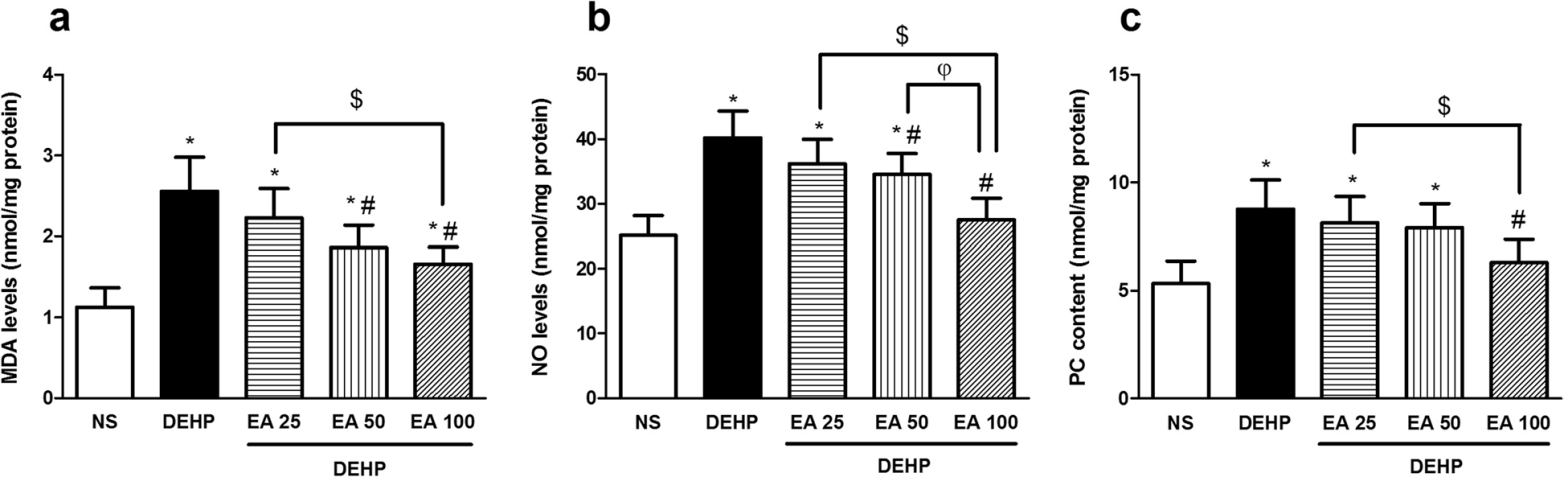
The effect of EA on MDA, PC and NO level in testes of mice exposed to DEHP. Values are means ± S.D. (*n* = 7). *p < 0.05 as compared to control group. ^#^*p* < 0.05 as compared to DEHP group

**Fig. 3:**
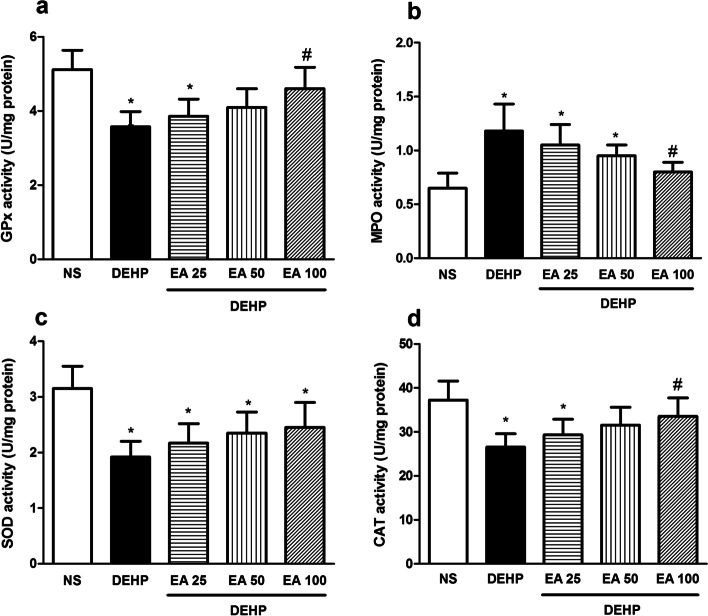
The effect of EA on GPx, SOD, CAT and MPO activity in testes of mice exposed to DEHP. Values are means ± S.D. (*n* = 7). *p < 0.05 as compared to control group. ^#^*p* < 0.05 as compared to DEHP group

Exposure of mice with DEHP significantly increased the activity of MPO and the level of MDA, PC and NO· compared to the control group (*p* < 0.05). Treatment with EA reduced DEHP-induced elevation of MDA and NO· in doses of 50 and 100 mg/kg (*p* < 0.05) and PC in the dose of 100 mg/kg (*p* < 0.05; Fig. 2). The activity of MPO reduced in animals treated with 100 mg/kg of EA compared to the DEHP group (*p* < 0.05; Fig. 3).

**Fig. 4:**
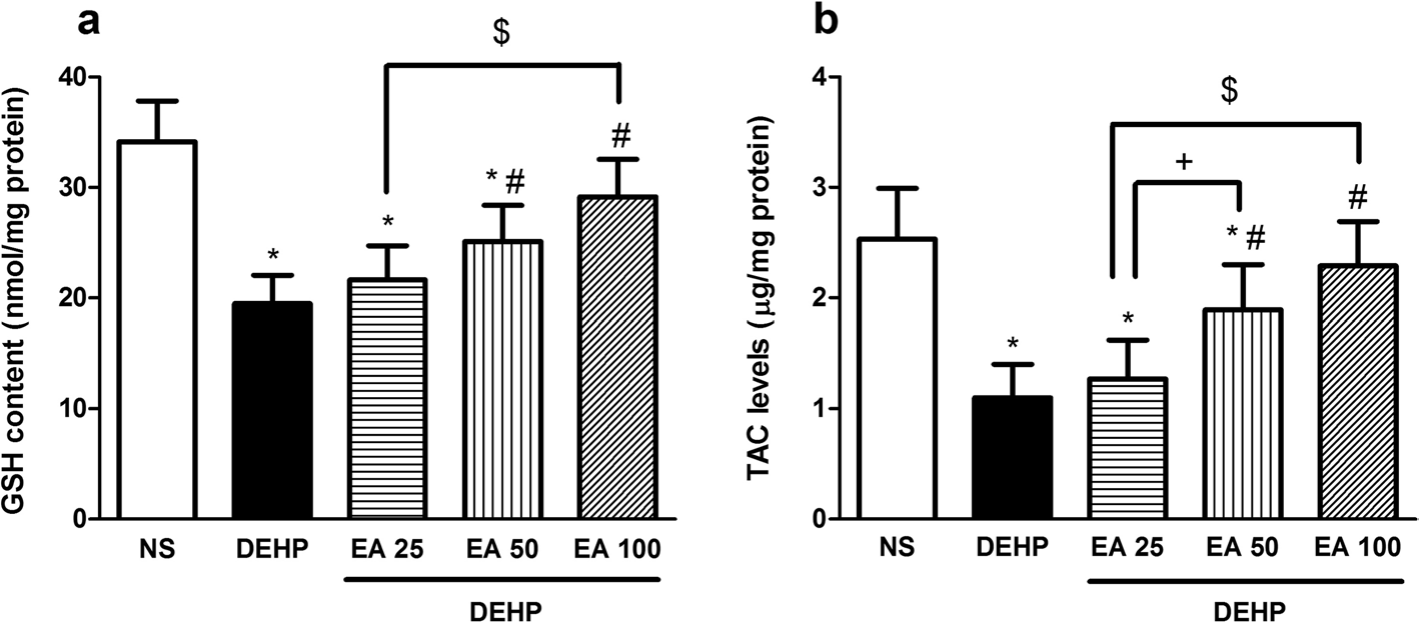
The effect of EA on GSH and TAC levels in testes of mice exposed to DEHP. Values are means ± S.D. (*n* = 7). *p < 0.05 as compared to control group. ^#^*p* < 0.05 as compared to DEHP group

Obtained results indicated that DEHP significantly decreased the activity of antioxidant enzymes (SOD, CAT and GPx) and the content of GSH and TAC in the testicular tissue in comparison with the control animals (*p* < 0.05). Treatment with EA in doses of 50 and 100 mg/kg significantly inhibited DEHP-induced reduction in SOD and GPx activity (*p* < 0.05). Treatment with EA could not inhibit DEHP-induced inhibition of CAT activity in the testicular tissue (Fig. 3) Results indicated that EA in the dose of 50 and 100 mg/kg significantly inhibited DEHP-induced reduction of GSH and TAC contents in the testicular tissue (*p* < 0.05; Fig. 4).

### Effects of EA on DEHP-induced inflammatory responses

**Fig. 5:**
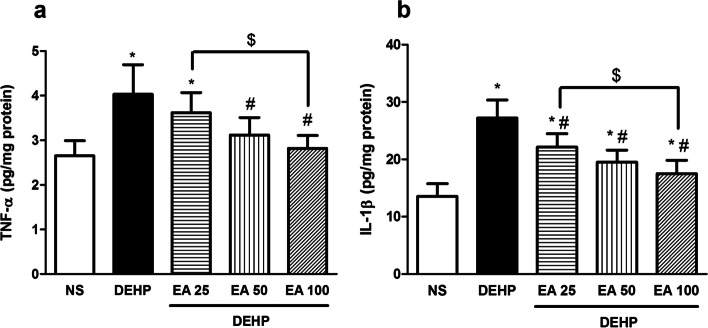
The effect of melatonin on TNF-α and IL-1β levels in testes of mice exposed to DEHP. Values are means ± S.D. (*n* = 8). *p < 0.05 as compared to control group. ^#^*p* < 0.05 as compared to DEHP group

Exposure of mice with DEHP resulted in the significant elevation in the expression of pro-inflammatory cytokines including TNF-α and IL-1β compared to the control group (*p* < 0.05). Administration of EA could significantly inhibit DEHP-induced increase in IL-1β levels in all doses (25, 50 and 100 mg/kg; *p* < 0.05). The level of TNF-α decreased in groups treated with 50 and 100 mg/kg of EA (*p* < 0.05) in comparison with DEHP group (Fig 5).

### Effects of EA on DEHP-induced alterations in histopathological parameters

**Fig. 6:**
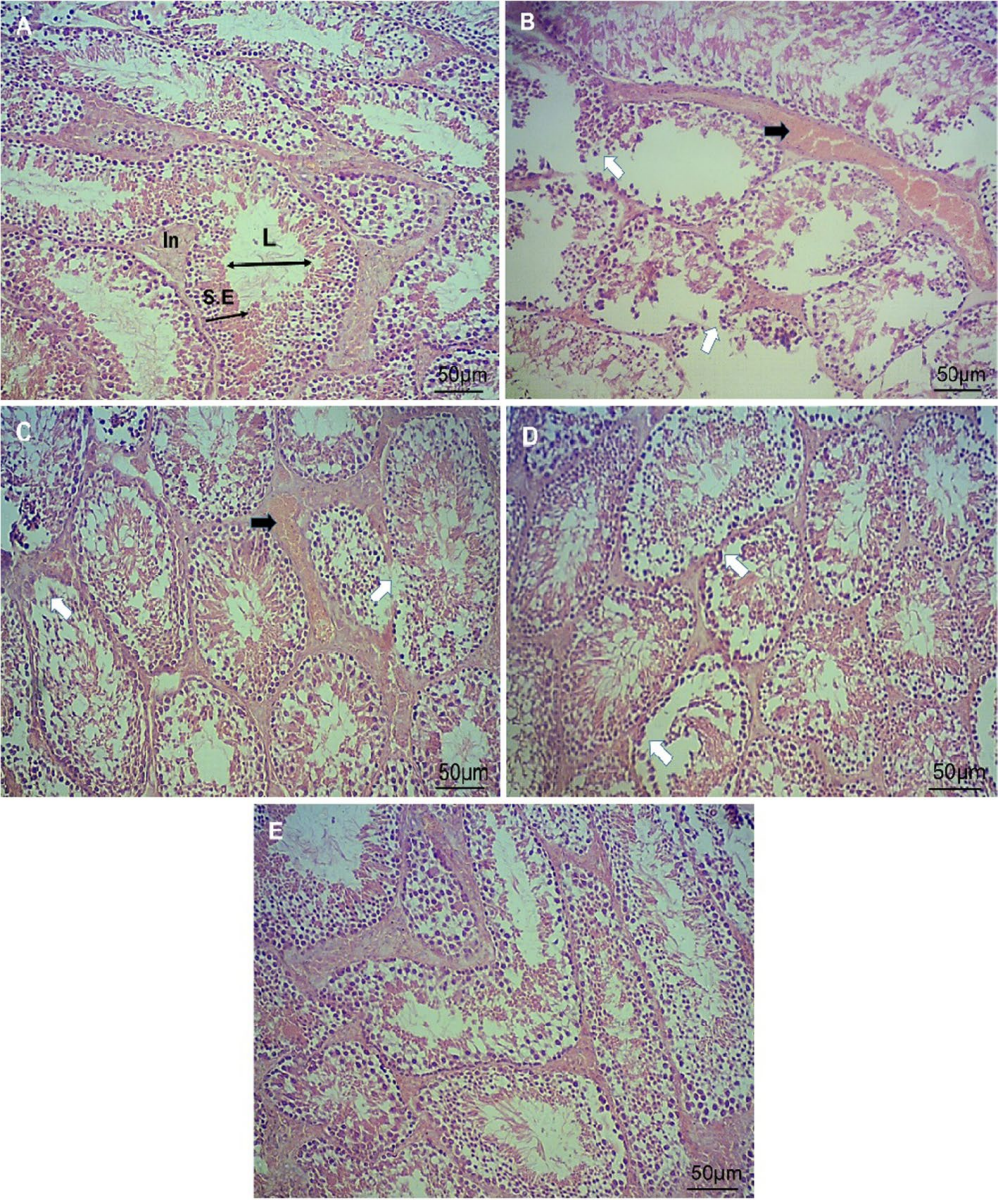
Cross section of testicular tubules (Magnification: 100 X) showing effects of EA on DEHP-induced testicular toxicity. **A** control group (**B**) DEHP group. **C** DEHP+EA (25 mg/kg/day) group. **D** DEHP+EA (50 mg/kg/day) group. **E** DEHP+EA (100 mg/kg/day) group. White arrows show the loss of germinal epithelium cells and black arrows show loss of spermatogenesis. Seminiferous epithelial; S. E, intertubular space; In and seminiferous lumen; L

Histological findings indicated the reduction in the number of germinal cells and damage in testicular structure (*p* < 0.05). Treatment with 25 mg/kg of EA remarkably inhibited DEHP-induced reduction of seminiferous epithelial height and number of spermatogonia, early spermatid and late spermatid, but did not show significant effect on seminiferous luminal and tubular diameters, tunica albuginea height and the number of primary spermatocyte. Treatment with 50 and 100 mg/kg EA significantly improved the number of germinal cells and structural damages of testes (*p* < 0.05); however, 50 mg/kg of EA could not improve tunica albuginea height (Table 2 and Fig. 6).

**Table 2:** Results of histopathological examination

	NS	DEHP	DEHP + EA 25	DEHP + EA 50	DEHP + EA 100
Tunica albuginea height (μm)	45.4 ± 5.2	19.1 ± 0.9 ^a^	21.1 ± 4.1 ^a^	24.5 ± 1.2 ^a^	43.6 ± 5.4 ^b, c, d^
Seminiferous tubular diameter (μm)	173.7 ± 34.1	80.0 ± 15.31 ^a^	97.0 ± 9.1 ^a^	137.0 ± 21.91 ^b, c^	151.6 ± 32.7 ^b, c^
Seminiferous luminal diameter (μm)	72.3 ± 6.1	41.6 ± 11.9 ^a^	47.0 ± 17.7 ^a^	73.5 ± 8.6 ^b, c^	88.9 ± 10.7 ^b, c^
Seminiferous epithelial height (μm)	71.3 ± 5.4	16.4 ± 1.8 ^a^	28.9 ± 2.7 ^a, b^	46.4 ± 2.78 ^a, b, c, d^	63.9 ± 3.2 ^a, b, c, d^
Number of spermatogonia /tubule	31.9 ± 3.6	11.1 ± 2.1 ^a^	23.3 ± 2.3 ^a, b^	27.4 ± 3.1 ^a, b, c, d^	31.6 ± 3.7 ^a, b, c, d^
Number of primary spermatocyte /tubule	45.3 ± 5.7	16.8 ± 3.6 ^a^	22.7 ± 4.2 ^a^	33.2 ± 4.8 ^a, b, c^	35.3 ± 5.1 ^a, b, c^
Number of early spermatid /tubule	53.9 ± 6.4	22.5 ± 3.3 ^a^	33.1 ± 3.7 ^a, b^	38.4 ± 4.2 ^a, b^	43.2 ± 5.2 ^a, b, c^
Number of late spermatid /tubule	59.3 ± 6.5	30.8 ± 4.8 ^a^	43.7 ± 5.3 ^a, b^	48.1 ± 5.5 ^a, b^	53.7 ± 6.1 ^b, c^

Values are means ± S.D. (*n* = 7)

^a^*p*<0.05 as compared to control group

^b^*p*<0.05 as compared to DEHP group

^c^*p*<0.05 as compared to DEHP+ EA 25 group

^d^*p*<0.05 as compared to DEHP+ EA 50 group

### Effects of EA on DEHP-induced alterations in sperm characteristics

The percentage of motile living sperm and sperm with progressive motility was significantly reduced in mice exposed to DEHP compared to the control group (*p* < 0.05). Treatment with 25 mg/kg EA significantly improved DEHP-induced changes in the number of motile living sperms (*p* < 0.05) but did not increase the number of progressive sperm. Treatment with 50 and 100 mg/kg EA significantly improved all sperm characteristics compared to DEHP treated mice (*p* < 0.05; Table 3).

**Table 3:** Results of sperm motility, progressive motility and vitality

variable		NS	DEHP	DEHP+ EA 25	DEHP+ EA 50	DEHP+ EA 100
Motility (%) Progressive (%)		78 ± 9 51 ± 7	42 ± 6 ^a^ 20 ± 3 ^a^	53 ± 4 ^a, b^ 24 ± 3 ^a^	58 ± 7 ^a, b^ 30 ± 5 ^a,b^	62 ± 6 ^a, b^ 35 ± 5 ^a, b, c^
Vitality (%)	Motile living sperm Immotile living sperm Dead sperm	43 ± 6 29 ± 4 25 ± 5	15 ± 2 ^a^ 41 ± 8 ^a^ 48 ± 6 ^a^	21 ± 3 ^a, b^ 37 ± 5 40 ± 7 ^a^	25 ± 4 ^a, b^ 33 ± 5 35 ± 3 ^a, b^	37 ± 3 ^a, b, c, d^ 30 ± 2 ^b^ 28 ± 4 ^b, c^

Values are means ± S.D. (*n* = 7)

^a^*p*<0.05 as compared to control group

^b^*p*<0.05 as compared to DEHP group

^c^*p*<0.05 as compared to DEHP + EA 25 group

^d^*p*<0.05 as compared to DEHP + EA 50 group

## Discussion

Environmental and occupational factors along with lifestyle practices have deleterious effects on semen quality and male reproductive health [17]. Di(2-ethylhexyl) phthalate is one of environmental factors with severe male reproductive system toxicity; DEHP causes testicular damage resulting in the reduction of testis weight, testosterone concentration and epididymal sperm counts. Since accessory sex organs are highly androgen-dependent, anti-androgenic effects of DEHP leads to the reduction of seminal vesicle and ventral prostate weights [18]. In line with these findings, our results showed that DEHP disrupted testicular function in mice characterized by the abnormality in the serum testosterone, FSH and LH level, reduction of germinal cells numbers and damage in testicular structure. Furthermore, DEHP affected the quality of sperms through reducing the sperm vitality and progressive motility. Seminiferous tubules include peritubular cells, germ cells and Sertoli cells. Germ cells give rise to the spermatogonia, spermatocytes, spermatids and spermatozoon through spermatogenesis process. Sertoli cells supports germinal cells in the spermatogenesis process [19]. Therefore, DEHP-induced disturbance in spermatogenesis results from not only direct injury to germ cells but also disruption of Sertoli cell function. The DEHP-induced reduction of testosterone generation results from the injury of Leydig cells; these cells are localized in the intertubular space and secrete testosterone; in response to the reduction of testosterone level, LH and FSH secretion increased as a positive feedback [20, 21]. Testicular cell damage induced by DEHP is associated with the mitochondrial dysfunction in, which this contributes to the excessive generation of ROS/RNS and induction of the mitochondrial apoptotic pathways; these events may finally contribute to testicular atrophy [22]. DEHP activates P53 signaling pathway in prepubertal testes, which this effect results in the enhancement of cell apoptosis and inhibition of proliferation of Leydig cells [23]; activation of P53 may play a major role to induce testis injury and reproductive dysfunction [24]. Elevated level of DEHP-induced oxidative stress is also associated with the reduction of the expression of PI3K, p-Akt and p-mTOR proteins, leading to the activation of the downstream autophagy-related proteins including ULK1, Beclin-1, autophagy-related gene 7 (Atg7), LC3-II. Autophagy activation may protect testes from DEHP-induced reproductive damage [24]. The reduction of sperm motility induced by DEHP may result from the impairment of ATP production; DEHP inhibits the expression of subunits of oxidative phosphorylation (OXPHOS) complex II, III, IV and V, which this effect is correlated with the attenuation of ATP levels in testes [22]. Since spermatozoa motility depends on sperm ATP content, DEHP-induced attenuation of ATP production may contribute to the male fertility problems related to the reduction of sperm motility [25].

Obtained results from our study indicated that EA could improve DEHP-induced changes in serum hormones levels, sperm motility, germinal cells numbers and testicular structure. Treatment of animals with EA ameliorated DEHP-induced MDA, NO˙, PC and MPO levels and increased the capacity of antioxidants. These results suggested that EA may improve testicular cell function through reducing DEHP-induced oxidative stress; these effects may inhibit the degeneration of Leydig and Sertoli cells leading to the improvement of testosterone level, and sperm count and motility. As a feedback response, enhancement of testosterone level lead to the reduction of LH and FSH levels. In line with our results, previous studies indicated that EA improve testicular function through reducing of oxidative stress. In monosodium glutamate-induced testicular injury, EA has been reported to enhance male reproductive capacity through elevation of enzymatic antioxidants levels and reduction of lipid peroxidation; this results in the elevation of testosterone hormone levels [26]. Furthermore, EA improves sodium arsenite-induced testicular toxicity by enhancing testicular antioxidant capacity leading to improvement of the serum testosterone level and testes histological parameters [27]. Ellagic acid has also been reported to reduce apoptosis in adriamycin-induced testicular toxicity, which this effect is associated with the reduction of oxidative stress; EA decreases the adriamycin-induced increase in the ratio of pro-apoptotic (Bax) and anti-apoptotic (Bcl-2) proteins resulting in the reduction of apoptotic cell death [28]. This antioxidant effect of EA results from direct scavenging of free radicals or indirect elevation of antioxidant capacity [27, 29]. Furthermore, results from previous studies indicate that EA activates autophagy in various pathological conditions [30], which this effect may be useful in reducing toxic effects of DEHP.

Exposure to DEHP has been reported to promote the expression of inflammatory mediators through inducing the nuclear translocation of the nuclear factor (NF)-κB; this effect results in the up-regulation of the expression of pro-inflammatory cytokines including IL-1β, IL-6, IL-8, and TNF-α [31]. Since oxidative stress induces NF-κB nuclear translocation [1], DEHP is suggested to induce inflammation in the testes through increasing ROS/RNS generation. Inflammation is one of factors leading to the male infertility through destruction of spermatogenesis [32]; TNF-α has been reported to negatively impact spermatozoa motility [33]. low dose of DEHP is suggested to be sufficient to promote induction of testis inflammation (orchitis) [34]. Our results indicated that EA inhibited DEHP-induced inflammatory responses testicular tissue of mice through down-regulating the expression of TNF-α and IL-1β. The anti-inflammatory effect of EA has also been reported by previous studies; EA attenuates inflammatory processes via inhibition of NF-κB pathway. Suppression of NF-κB activation in turn leads to the reduction of the expression of inflammatory cytokines such as IL-1β and TNF-α [35].

## Conclusions

This research revealed that EA may be useful agent to reduce DEHP-induced testicular toxicity. The testicular toxicity od DEHP is associated with the excessive level of oxidative/nitrosative stress and induction of inflammatory responses; These contribute to the alteration in the serum level of testosterone, LH and FSH, sperm characteristics and spermatogenic cells numbers. From these findings, it is suggested that therapeutic agents with antioxidant and anti-inflammatory effect may inhibit DEHP-induced gonadotoxicity. Our results showed that EA could prevent the testicular toxicity of DEHP through inhibition of oxidative/nitrosative stress and inflammatory responses, which these effects were associated with improvement in the testicular structure, sperm motility and number, and hormone levels. Our data raise the possibility that EA may be beneficial to inhibit male reproductive toxicity induced by endocrine disrupting chemicals such as DEHP.

## Data Availability

The datasets used and/or analysed during the current study are available from the corresponding author on reasonable request.
